# Opening the Special Issue “Emerging Public Health Challenges and Healthcare Preparedness: Evidence-Informed Perspectives for Prevention, Response, and Risk Communication”

**DOI:** 10.3390/healthcare14152287

**Published:** 2026-07-27

**Authors:** Marco Dettori

**Affiliations:** Department of Medicine, Surgery and Pharmacy, University of Sassari, 07100 Sassari, Italy; madettori@uniss.it

## 1. Why Timely, Evidence-Informed Perspectives Matter for Public Health Preparedness

Public health professionals and healthcare systems increasingly operate within a compressed time frame. New threats emerge rapidly, established risks change their geographical and social distribution, and information environments can amplify uncertainty before institutions have completed a full scientific assessment. They are therefore required to interpret emerging threats in real time, communicate uncertainty transparently, and translate evolving evidence into preparedness actions before crises are fully defined. In this setting, the quality of interpretation becomes as important as the speed of response. In this context, a curated collection of concise, evidence-informed perspectives is particularly timely.

Preparedness must be understood as more than emergency response. It includes surveillance, early warning, workforce protection, continuity of essential services, laboratory and referral capacity, risk communication, community engagement, governance, and the ability to learn before and after crises. The World Health Organization frames preparedness, response, resilience, and community protection as connected functions rather than separate stages [1,2]. The WHO Pandemic Agreement further reinforces the importance of equity, cooperation, and shared responsibility in pandemic prevention, preparedness, and response [3].

This broader understanding changes how healthcare systems should interpret emerging threats. Outbreaks, climate-related emergencies, misinformation, and digital innovation each test whether systems can convert evidence into coordinated, trusted, and equitable action. The Special Issue will therefore address a deliberately broad but coherent set of themes, including emerging and neglected infectious diseases, healthcare-system readiness and resilience, environmental and climate-related health emergencies, surveillance and early warning, risk communication and public trust, vulnerable populations, migration and mobility, digital health and artificial intelligence, and healthy and resilient healthcare environments.

Risk communication and community trust deserve particular attention. Public health measures depend on cooperation, and cooperation depends on credibility. Information alone is insufficient when communities do not trust the source, when uncertainty is concealed, or when communication ignores fear, stigma, culture, and lived experience. Effective preparedness must therefore integrate communication and engagement before emergencies, not add them after technical decisions have already been made [4]. Equity is equally central. Frontline communities and health systems often bear the greatest burden of detecting, containing, and responding to threats that have wider regional or global relevance. Preparedness that depends on late external attention is both ethically weak and operationally inefficient. Support for local capacity, workforce protection, essential services, and community engagement should begin before escalation.

Technology will also be examined critically. Artificial intelligence, digital surveillance, telemedicine, predictive modelling, and data platforms can strengthen preparedness, but only when they improve real decisions and local capacity. A technically sophisticated tool that cannot be implemented, trusted, maintained, or acted upon does not constitute preparedness. Innovation should therefore be judged by whether it makes systems faster, safer, clearer, more equitable, and more usable.

## 2. A Curated Space for Authoritative Public Health Perspectives

This Special Issue, “Emerging Public Health Challenges and Healthcare Preparedness: Evidence-Informed Perspectives for Prevention, Response, and Risk Communication”, is intended to create a curated space for concise, authoritative, and evidence-informed perspectives on issues that require timely public health attention.

These contributions are not intended to replace systematic reviews, original studies, formal guidelines, or institutional risk assessments. Their function is different. A well-constructed perspective should explain why an issue matters now, distinguish established knowledge from uncertainty, identify the most relevant implications for healthcare systems and public health practice, and provide a balanced framework for action. The value of this format lies not in presenting every available fact but in selecting the facts that matter, clarifying their significance, and indicating where action, caution, or further evidence is needed.

The ambition of this Special Issue is modest in format but substantial in purpose. It seeks to offer readers clear, timely, and useful scholarly interpretation without alarmism or overstatement. Emerging public health challenges will continue to test healthcare systems before the evidence is complete and before political attention is guaranteed. In that environment, rigorous and proportionate interpretation is itself a preparedness function. By bringing together focused and evidence-informed perspectives, this Special Issue aims to support that function and to encourage further contributions that strengthen preparedness, prevention, response, and risk communication in the face of emerging public health challenges.
